# Massive suprachoroidal hemorrhage: Surgical management and outcome

**DOI:** 10.3205/oc000032

**Published:** 2015-10-23

**Authors:** Thomas Laube, Claudia Brockmann, Norbert Bornfeld

**Affiliations:** 1 Centre for Ophthalmology Düsseldorf, Germany; 2 Department of Ophthalmology, University Hospital, University of Duisburg-Essen, Essen, Germany

**Keywords:** massive suprachoroidal hemorrhage, cataract extraction, vitrectomy, silicone oil

## Abstract

**Objective:** To describe options for vitreoretinal surgery in the management of massive suprachoroidal hemorrhage (SCH).

**Methods:** Visual acuity (VA), ocular findings, timing of surgical intervention, surgical procedures, and outcomes of four patients diagnosed with massive SCH and admitted to the University Eye Clinic Essen were reviewed retrospectively.

**Results:** Four eyes of four patients (mean age, 82 years; range, 74–89 years) were studied. In three cases the occurrence of SCH was related to cataract surgery and occurred intra- or postoperatively. One patient developed spontaneous SCH of unclear origin. Three patients had a history of arterial hypertension; one eye had high myopia, two patients suffered from cardiovascular diseases, and two patients had glaucoma. Postoperative follow up of the patients ranged from 5 to 29.5 months (mean, 19.6 months). Transscleral drainage of SCH was in all cases combined with pars plana vitrectomy, use of heavy liquids (perfluorodecalin) and silicone oil tamponade. The mean time interval from hemorrhage to surgical intervention was 16.5 days (range 5–29 days). Preoperative VA of all eyes was light perception. Two patients achieved a final postoperative visual acuity of 20/20 and 20/320, respectively, one patient improved to hand motion, and one patient resulted in no light perception.

**Conclusions:** Surgical interventions including transscleral drainage of SCH, vitrectomy, and silicone oil tamponade are valuable options in the management of massive SCH to save the eye and possibly improve the otherwise extreme poor prognosis.

## Introduction

Massive suprachoroidal hemorrhage (SCH) is a rare sight-threatening complication associated with incisional intraocular surgery [1], [2], [3], [4]. SCH may occur during several surgical procedures that result in significant fluctuations of the IOP (intraocular pressure) [5]. The intraoperative acute hypotony is the triggering factor causing changes of the transluminal vascular pressure that may result in choroidal effusion with possible rupture of small arteries crossing the suprachoroidal space [6].

Suprachoroidal hemorrhage has been reported in association with cataract surgery [2], [7], [8], [9], glaucoma filtering procedures [10], keratoplasty [11], [12], and vitreoretinal surgery [13], [14]. The reported incidence of SCH during or after cataract surgery ranges from 0.03% to 0.13% [2], [7], [15]. Extracapsular cataract extraction (ECCE) resulted in a higher incidence of SCH (0.28%, [16]) in comparison to phacoemulsification [2], [8]. In phacoemulsification procedures the self-sealing corneal incision enables rapid closure of the wound and provides effective tamponade against the development of SCH [7]. For pars plana vitrectomy incidence rates for SCH of 0.4% to 0.6% have been reported [17]. The incidence of expulsive SCH during glaucoma filtering surgery is approximately 0.15% [12]. 

Systemic risk factors associated with SCH are advanced age, systemic hypertension, arteriosclerosis, blood coagulation defects, intraoperative tachycardia, and diabetes mellitus [18]. Ocular risk factors for the development of SCH during intraocular procedures comprise glaucoma, elevated IOP, high myopia, aphakia, and pseudophakia [9], [10], [14], [16], [19]. The surgical management of SCH includes drainage through posterior sclerotomies, often combined with vitrectomy, and silicone oil instillation [3], [7], [18]. As most studies published to date are limited and report poor functional outcomes despite surgical intervention [3], [7], [19], [20], the present study focuses on timing, surgical management, and anatomic and functional results of four cases of massive SCH.

## Case descriptions

The records of 16.575 eyes from 9.371 patients undergoing cataract surgery between April 2004 and June 2014 at the Centre for Ophthalmology Düsseldorf were reviewed for the occurrence of SCH. One patient experienced intraoperative massive SCH in one eye and was transferred to the tertiary referral centre University Eye Clinic Essen (case 1). The review of patient records at the University Eye Clinic Essen within the corresponding period showed that three further patients were referred from three other centers for the treatment of massive SCH (cases 2–4). The surgical management and outcome of these four cases are described. Patient data included age, gender, known risk factors for SCH, the onset of SCH, the timing and performance of surgical intervention, the anatomic success, and the occurrence of complications. The preoperative lens status, the pre- and postoperative ocular findings and anatomy, the initial and final visual acuity (VA), and the duration to final follow-up were evaluated as well. 

Vitreoretinal surgery was performed in all cases under general anesthesia. In principle surgical intervention included placement of an infusion line into the anterior chamber and into the vitreous cavity whenever possible. Deep cut down sclerotomies were placed at the location of the most excessive choroidal detachment. Drainage was performed using a bent cannula and indentation of the peripheral choroid. This process was supported by injection of sodium hyaluronate viscoelastic solution (Provisc^®^, Alcon Pharma GmbH) posterior to the intraocular lens (IOL) whenever accessible to further flatten the choroidal detachment. Complete pars plana vitrectomy was performed followed by instillation of perfluorocarbon liquid (PFCL, DECALIN^®^, Fluoron GmbH) into the vitreous cavity, resulting in further drainage of suprachoroidal blood. PFCL was then exchanged to silicone oil (SIL-OL^®^ 5000, Acri.Tec GmbH) in all eyes continuing drainage of suprachoroidal blood through the sclerotomies as complete as possible. Finally, the infusion cannula was removed, the sclerotomies were closed using 7×0 polydioxanone interrupted sutures, and the conjunctiva was closed using 7×0 polyglactin interrupted sutures. The main steps of the surgical procedure as performed in case 2 are shown in the video file (Attachment 1) supplemented to the online version of the article. This study was carried out in accordance with the principles of the Declaration of Helsinki.

Four eyes of four female patients with SCH were reviewed. The mean age of the patients was 82 years and ranged from 74 to 89 years. The clinical data of the cases are summarized in Table 1 (Tab. 1). In each case one to five risk factors for the development of SCH were present. Three cases had arterial hypertension, two cases had atrial fibrillation, and two cases had glaucoma. One case had high myopia and another case had tachycardia, absolute arrhythmia, and aortic sclerosis. All patients were on long term anticoagulation therapy with acetylsalicylic acid or phenprocoumon at the time of SCH. 

All eyes underwent previous cataract extraction and subsequent placement of a posterior chamber lens. On initial examination cases 1, 2, and 4 showed centered IOLs, case 3 presented with a dislocated IOL. The SCH was related to cataract surgery in three cases and occurred intraoperatively (case 1), 24 hours (case 2) or 3 weeks (case 3) postoperatively (Table 1 (Tab. 1)). In case 1 the SCH occurred after implantation of the IOL and an immediate tamponade was achieved by suturing all surgical incisions. Case 4 developed spontaneous SCH of unclear origin. This patient underwent cataract surgery three years previously. In all cases the SCH was massive with pronounced choroidal detachment forming kissing choroids. 

**Figure 1:**
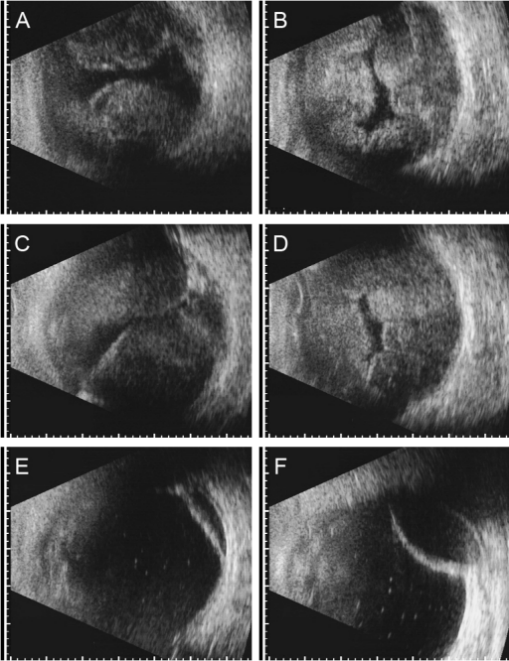
Case 1, transverse B-scan ultrasonography of the left eye. A, B Six days preoperatively. Massive suprachoroidal hemorrhage with circular kissing choroids. Visual acuity was hand motion. A from inferior. B from temporal. C, D Two days preoperatively. The ultrasonic findings deteriorated, the vitreous cavity was increasingly constricted. Visual acuity was light perception. C from inferior. D from temporal. E, F 14 months postoperatively, six days after removal of silicone oil. The choroid was widely reattached, a limited choroidal detachment persisted in the temporal and nasal periphery. Visual acuity was 20/80. E from nasal. F from temporal

Figure 1 A, B (Fig. 1) shows the ultrasonic findings in case 1 three days after onset of a massive SCH with circular kissing choroids resulting in a VA of hand motion. Following the patient for four days showed further progression of the choroidal detachment resulting in apposition of the bullous choroidal detachment (Figure 1 C, D (Fig. 1)). Visual acuity decreased to light perception. The patient underwent subsequent surgical treatment.

Surgical intervention was performed in all cases 5–29 days (mean 16.5 days) following onset of SCH (Table 1 (Tab. 1)). All patients underwent transscleral suprachoroidal drainage procedures with pars plana vitrectomy, use of heavy liquids (PFCL) to displace the hemorrhage from the posterior pole, and silicone oil tamponade. A complete drainage of the hemorrhage was possible in case 2. In cases 1 and 3 most of the SCH could be removed. In case 4 a major SCH persisted. Complete resolution of the choroidal detachment could be achieved in case 2. In case 1 four-fifths and in case 3 two-thirds of the choroid were completely drained. In case 4 the bullous choroidal detachment could only be reduced by the surgical intervention. In case 3 the IOL was temporally subluxated and part of the vitreous prolapsed into the anterior chamber. The IOL was extracted from the anterior chamber using a scleral tunnel.

**Figure 2:**
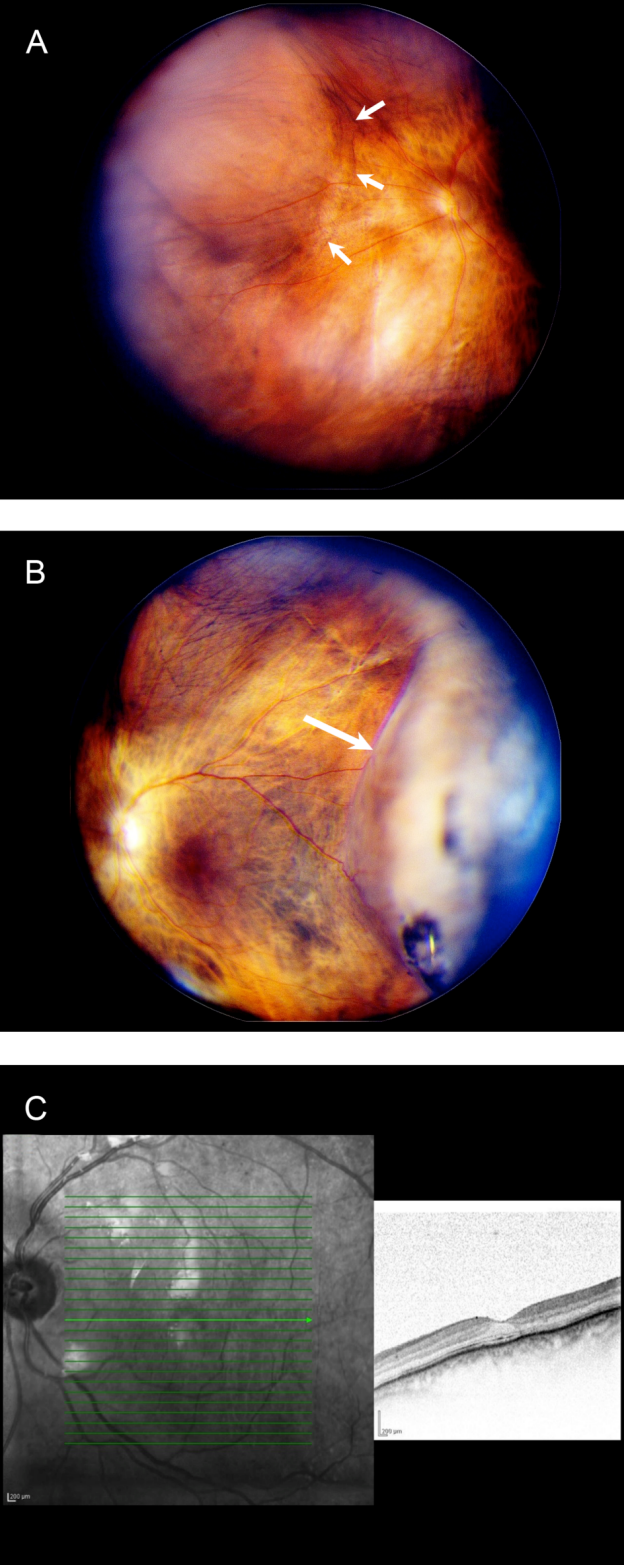
Fundus views of case 1 (left eye), 6 months after surgical treatment of suprachoroidal hemorrhage (sclerotomies, vitrectomy, silicone oil instillation). The best corrected visual acuity was 20/30. A From nasal. The previously circular choroidal detachment was regressive, its former extension lines were visible as pigment deposits in the nasal area (arrows). B Infero-temporally a choroidal amotio remained (arrow). C Optical coherence tomography of the intact foveal region

Figure 2 (Fig. 2) illustrates the ophthalmoscopic findings of case 1 six months postoperatively. The previously circular choroidal detachment was regressive and its former extension lines were visible as pigment deposits in the nasal area (Figure 2 A, arrows (Fig. 2)). Infero-temporally residual choroidal detachment persisted (Figure 2 B (Fig. 2)). The anatomy of the posterior pole was preserved as demonstrated in the optical coherence tomography (Figure 2 C (Fig. 2)). The VA at this stage was 20/30. Figure 1 E, F (Fig. 1) shows the ultrasonographic findings of case 1 fourteen months postoperatively and six days after removal of the silicone oil. The choroid was mostly reattached. A limited choroidal detachment persisted in the temporal and nasal periphery. The VA at this time was 20/80.

Patients were observed from 5 to 29.5 months (mean, 19.6 months) postoperatively. The preoperative VA was in all cases light perception. The best VA in the follow-up period was 20/20 (case 1), 20/320 (case 2), counting fingers (case 3), and light perception (case 4), respectively. The visual acuities at last follow-up (Table 1 (Tab. 1)) equaled the best VA in cases 1 and 2; a decrease from best to final VA of one line was noticed in cases 3 and 4. Thus a visual improvement was achieved in cases 1, 2, and 3, while the visual acuity decreased from light perception to no light perception in case 4.

Patient 1 developed glaucoma 4.5 months postoperatively (24 mmHg) and received topical medical treatment resulting in a final IOP of 16 mmHg. Patient 4 showed an IOP increase of up to 50 mmHg five days postoperatively that was lowered under systemic medication. Subsequently this patient had a recurrent SCH, prolapse of silicone into the anterior chamber and blood within the silicone filling. No further surgical intervention was performed in this case. At final presentation 2.5 years postoperatively the IOP was 17 mmHg and the visual acuity was no light perception.

A postoperative IOP rise was also noticed in patient 3; an IOP of 44 mmHg three days postoperatively required topical treatment and intravenous therapy resulting in an IOP of 17 mmHg at final follow-up. This patient was diagnosed with glaucoma at first presentation and continued with the previous glaucoma therapy. Patient 2 showed no complications during the follow-up period.

The anatomic result at final examination was an attached retina and persisting peripheral choroidal detachment in case 1, an entirely attached retina in case 2, and an attached retina with a large central whitish scar in case 4. In case 3 the retina was attached under silicone oil, the choroidal detachment was resolved. As the optic nerve head showed incipient atrophy no further treatment was performed in this case. The silicone oil was removed in case 1 fourteen months postoperatively. In the other patients the silicone oil was not removed.

## Discussion

Suprachoroidal hemorrhage is a disastrous but rare complication of intraocular surgery. Kuhn et al. analyzed seven reports on SCH and found that untreated eyes with a slightly delayed wound closure became phthisical or were eviscerated/enucleated in 43%; 41% of the eyes had no light perception or light perception vision, and only 11% showed greater than count finger vision [21]. 

The present study describes the result of a retrospective study on four cases of intraoperative, postoperative, and spontaneous SCH. The patients had one to five systemic or ocular risk factors for developing SCH. The systemic risk factors were hypertension, aortic sclerosis, and tachycardia. Glaucoma and high myopia were additional ocular risk factors. These findings are consistent with other studies [5], [9], [16], [20]. Further risk factors for the development of SCH are advanced age and a history of previous vitrectomy [7]. Aphakia and pseudophakia have been identified as risk factors for SCH as well [14], [19]. Our study confirms this observation as all patients were pseudophakic. Three patients presented with a combination of multiple risk factors (Table 1 (Tab. 1)), supporting a multifactorial pathophysiological mechanism of SCH [3]. 

While in cases 1–3 the SCH was related to cataract surgery, case 4 developed a spontaneous SCH, cataract surgery was three years previously. At presentation, a suprachoroidal and subretinal hemorrhage was diagnosed and confirmed during surgery. In this patient, a temporary inadequate anticoagulant medication resulted in an INR (International Normalized Ratio) value of up to 5, substantially differing from the therapeutic target. It can be assumed that this condition was the trigger for the occurrence of pronounced SCH. An association of massive spontaneous SCH with the use of systemic anticoagulants or thrombolytic agents in the absence of surgery or trauma has been previously reported in a number of case studies [22], [23]. Moreover, all other patients of our study were on long term anticoagulation therapy at the time of SCH, as well (Table 1 (Tab. 1)).

Despite the poor visual outcome of no light perception in case 4, the anatomic outcome at final presentation (29.5 months postoperative) was good showing a reattached choroidea and retina following absorption of the hemorrhage. Excellent final anatomic results were also achieved in cases 1 and 2 and in subtotal regression of the choroidal detachment in case 3.

Preoperatively all four patients of our study had a VA of light perception, at final presentation the VA improved in three patients, only in case 4 light perception was lost. Incipient optic nerve atrophy and the large central chorioretinal scar were the cause for the poor visual outcome in this patient. While the VA of case 3 improved to hand motions, case 2 had a better result with a final VA of 20/320. The excellent functional outcome of 20/20 in case 1 is remarkable and differs from other studies that report a generally poor functional outcome after surgical treatment of massive SCH [3], [7], [18], [20], [23], [24]. Patient 1 was the only case of intraoperative SCH in our study and an immediate tamponade was performed by fast suturing of all surgical incisions. 

We performed pars plana vitrectomy, posterior drainage sclerotomies, and tamponade with silicone oil instillation in all cases. A silicone oil tamponade has been shown to be advantageous over balanced salt solution or gas filling as it protects against choroidal re-bleeding and prevents the development of chronic hypotony [3], [25]. While none of our patients developed postoperative hypotony, other studies report a frequency of hypotony ranging from 24% to 71% [1], [5], [13], [24]. The silicone oil tamponade was removed in case 1 fourteen months postoperatively without any complications, but remained in all other patients. Consistent with Meier and Wiedemann we recommend removing silicone oil only in eyes where a good visual function has been achieved or in cases where complications related to the silicone oil occur [3]. 

Our results show that the surgical management of massive SCH is effective to save the eye and to possibly achieve useful vision. The spontaneous course would have resulted in blindness in our cases. Several studies have shown that SCH is likely to result in phthisis and complete loss of vision if untreated [13], [18]. Meier and Wiedemann suggested to perform vitreoretinal surgery only in cases where the SCH extends to more than two quadrants posterior to the equator without involvement of the macula and in all eyes where the macula is affected by the hemorrhage [3]. Additionally, cases with kissing choroids, or cases with vitreous hemorrhage, vitreous incarceration or retinal detachment require surgical treatment [3]. However, smaller and limited suprachoroidal hemorrhages may resolve spontaneously [10]. 

The optimal timing for surgical treatment of SCH has not yet been defined [18]. In our study the mean time from the occurrence of SCH to surgical intervention was 16.5 days (range 5–29 days). In other studies the mean time interval was 11 days and a similar range (6–20 days) [3], [18]. Generally a longer duration of appositional SCH has been described to result in a poor visual outcome [24]. In our cases the worst visual outcome was noted after a time interval of 29 days between SCH and surgery (case 4, VA: no light perception, Table 1 (Tab. 1)), while the best final VA was achieved after a period of 9 days (case 1, VA: 20/20). Referring to these results an interval of 7 days may be optimal in which a clot lysis develops that facilitates successful transscleral drainage. In accordance with previous studies, the data of this study suggest that the time interval should not exceed 14 days [3], [24]. 

The first author reported an incidence rate for SCH of 0.006% (one case in 16.575 cataract surgeries performed in a period of 10 years). Compared to other studies, giving incidence rates of 0.03% to 0.13% [2], [7], [15], this is a significant lower rate. One possible reason for this difference is the exclusive performance of phacoemulsification procedures in all cases treated by the first author as compared to the literature data, where different surgical approaches (intracapsular cataract extraction, ECCE, phacoemulsification) have been analyzed. Lower incidences of SCH in phacoemulsification as compared with ECCE have been reported [2], [7]. 

Prophylaxis of expulsive SCH during open globe surgery is the priority objective requiring careful consideration of all risk factors and corresponding planning of the surgical procedure avoiding intraoperative hypotony as much as possible [21].

## Conclusion

This study has shown that massive SCH should be treated by a combined surgical approach including posterior sclerotomies, vitrectomy, and silicone oil instillation in order to preserve the globe and to prevent the otherwise devastating functional outcome and potential phthisis. Surgery should not be delayed more than 7 to 14 days after onset of SCH.

## Notes

### Competing interests

The authors declare that they have no competing interests.

## Supplementary Material

Video: Massive suprachoroidal hemorrhage: Main steps of the surgical management performed in case 2

## Figures and Tables

**Table 1:**
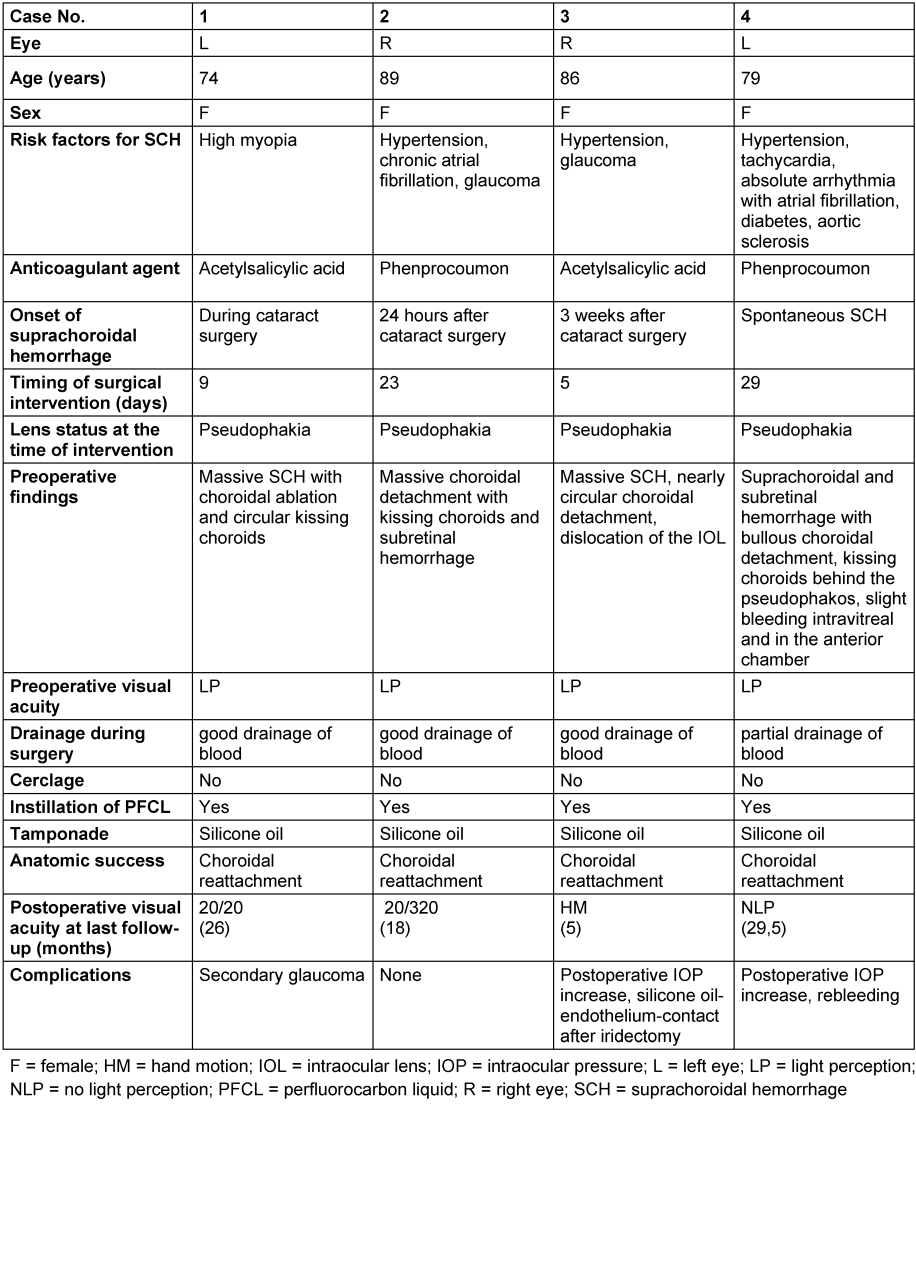
Clinical characteristics of patients with suprachoroidal hemorrhage
